# Semantic relatedness and similarity of biomedical terms: examining the effects of recency, size, and section of biomedical publications on the performance of word2vec

**DOI:** 10.1186/s12911-017-0498-1

**Published:** 2017-07-03

**Authors:** Yongjun Zhu, Erjia Yan, Fei Wang

**Affiliations:** 1 Healthcare Policy and Research, Weill Cornell Medicine, Cornell University, New York, NY USA; 2 College of Computing and Informatics, Drexel University, Philadelphia, PA USA

**Keywords:** Word2vec, Biomedical publications, PubMed, PubMed Central, Semantic similarity, Semantic relatedness

## Abstract

**Background:**

Understanding semantic relatedness and similarity between biomedical terms has a great impact on a variety of applications such as biomedical information retrieval, information extraction, and recommender systems. The objective of this study is to examine word2vec’s ability in deriving semantic relatedness and similarity between biomedical terms from large publication data. Specifically, we focus on the effects of recency, size, and section of biomedical publication data on the performance of word2vec.

**Methods:**

We download abstracts of 18,777,129 articles from PubMed and 766,326 full-text articles from PubMed Central (PMC). The datasets are preprocessed and grouped into subsets by recency, size, and section. Word2vec models are trained on these subtests. Cosine similarities between biomedical terms obtained from the word2vec models are compared against reference standards. Performance of models trained on different subsets are compared to examine recency, size, and section effects.

**Results:**

Models trained on recent datasets did not boost the performance. Models trained on larger datasets identified more pairs of biomedical terms than models trained on smaller datasets in relatedness task (from 368 at the 10% level to 494 at the 100% level) and similarity task (from 374 at the 10% level to 491 at the 100% level). The model trained on abstracts produced results that have higher correlations with the reference standards than the one trained on article bodies (i.e., 0.65 vs. 0.62 in the similarity task and 0.66 vs. 0.59 in the relatedness task). However, the latter identified more pairs of biomedical terms than the former (i.e., 344 vs. 498 in the similarity task and 339 vs. 503 in the relatedness task).

**Conclusions:**

Increasing the size of dataset does not always enhance the performance. Increasing the size of datasets can result in the identification of more relations of biomedical terms even though it does not guarantee better precision. As summaries of research articles, compared with article bodies, abstracts excel in accuracy but lose in coverage of identifiable relations.

## Background

Measuring semantic relatedness and similarity between biomedical terms is a classic research problem in the biomedical domain [1, 2]. Pedersen and colleagues defines semantic relatedness and similarity as “*Semantic relatedness is a more general notion of the relatedness of concepts, while similarity is a special case of relatedness that is tied to the likeness (in the shape or form) of the concepts.”* [1]. For instance, diabetes and insulin are related terms while glucose and blood sugar are similar terms. Measuring semantic relatedness and similarity has a broad impact on a variety of applications such as information retrieval, information extraction, and recommender systems. The most widely used approach to solving this problem is to utilize existing ontologies and thesauri such as UMLS and WordNet [3–5]. One strength of this approach is its reliability because it relies on verified human-built resources that make use of crowd intelligence. However, one limitation is its lack of adaptability to the changing research landscape because it largely depends on structure of ontologies [6]. Even though the limitation might be partially overcome by updating ontologies periodically, it requires substantial human resources and time. Another approach is the word embedding technique [7, 8]. The word embedding technique has been introduced and used for more than one decade to help understand words and their relations across corpora. It does not require any human intervention and automatically generates word similarity information from any given corpora. Compared to ontology- and thesaurus-based approaches, the word embedding technique has the following strengths: 1) it does not require human involvement, thus saving time and resources; 2) it can analyze and produce results from big data that humans are unable to; and 3) up-to-date results are available by feeding up-to-date corpora.

In recent years, we have witnessed a steady increase of biomedical publications. MEDLINE indexed less than 700,000 journal articles in 2005, but that number exceeded one million in 2015. Given that these publications contain rich biomedical knowledge, utilizing publication data to address a variety of biomedical problems is a promising approach adopted in previous studies [9, 10]. Due to large size of data, the word embedding technique is an ideal approach to get insights from biomedical publications. Among many algorithms of the word embedding technique, word2vec [7, 8], introduced in 2013, has been one of the most popular methods due to its sound performance. Despite its advantages, its applicability to the biomedical publications has not been fully studied. Therefore, in this paper, we aim to explore word2vec on biomedical publications and understand its ability of deriving semantic relatedness and similarity between biomedical terms. Our objectives are to explore how different settings on publication data affect the method’s performance: including the recency of publications, the size of publications, and the choice of different sections (abstracts vs. full texts). Results from this study will help biomedical researchers apply word2vec in a more informed way and improve the method’s performance on extracting similarity and relatedness information from biomedical publication data.

### Related work

In this section, we first briefly introduce word2vec and then survey the related work that used word2vec on biomedical publications. These studies primarily focused on the effects of architectures and parameter settings on experimental results. A few empirical studies were identified on how to configure the method to get better performance.

### Word2vec

Word2vec [7, 8] takes a corpus as input and produces a vector space. It estimates continuous vector representations of words from large corpora. Each unique word in the corpus is represented as a vector and words that share common contexts are positioned closely to each other. Similarity of two words are determined by calculating cosine similarity of two vectors that represent the two words. It was built on previous work on neural net language models (NNLM) and the advantage of word2vec is that it significantly reduced the computational complexity. Two word2vec models were proposed, one is the continuous bag-of-words (CBOW) model that does not consider word orders and the other is the continuous skip-gram model that assigns different weights based on the proximity of words in a window. Another well-known word embedding technique is GloVe [11] which was proposed by Pennington and colleagues. However, there are limited studies on applications of Glove in the biomedical domain. Readers can refer to [12] for technical differences between word2vec and GloVe.

Word2vec has been applied to textual data from a variety of sources such as social network services, online reviews, and scientific publications. It has been employed to address problems such as information retrieval, document categorization, sentiment analysis, and citation analysis. Amer and colleagues [13] proposed a word2vec-based method for efficient information retrieval. Word2vec was used to select additional terms that are related to query terms. Additional terms were then used to expand queries and retrieve more relevant documents. Ju and colleagues [14] employed word2vec and proposed a method of document categorization. Word vectors of words obtained from word2vec were multiplied with the words’ TF-IDF weighting to represent documents. Word2vec has also been used for sentiment classification. In a recent study [15], word2vec was used to cluster similar features reside in online product reviews, in which semantic distance between two features were obtained from their cosine similarity in the trained word2vec model. Word2vec model trained on microblog data has also been used to build a sentiment dictionary [16]. Jeong and Song [17] proposed a word2vec-based author similarity measure. Co-cited authors were identified in citing sentences and word2vec was applied to those citing sentences to calculate author similarity.

### Applications of Word2vec on biomedical publications

Minarro-Giménez and colleagues [18, 19] applied word2vec to PubMed abstracts to study the method’s ability of identifying relations between pharmaceuticals and diseases. This is one of the first studies that explored the performance of word2vec on biomedical text corpora for the problem of measuring relatedness of biomedical terms. The authors found that between the two model architectures of word2vec, the continuous skip-gram model performed better than CBOW. Additionally, the combination of 10 window size and 300 vector dimensions produced the best results and a window size greater than 20 and a vector dimension greater than 800 resulted in the deterioration of accuracy.

Muneeb and colleagues [20] explored word2vec on a dataset that includes 1.25 million PubMed Central (PMC) full-text articles. Both skip-gram and CBOW were tested with a few hyper-parameter settings on vector dimension (i.e., 25, 50, 100, and 200) and windows size (i.e., 9). For evaluation, two standard datasets were used: UMNSRS (University of Minnesota Semantic Relatedness Set)-Rel and UMNSRS-Sim, both created by Pakhomov and colleagues [21] at University of Minnesota. The two datasets were created by asking eight medical residents to judge 724 pairs of medical terms and include 587 and 566 pairs respectively. The best results obtained were correlation scores of 0.52 and 0.45 for semantic relatedness and similarity. Results showed that the skip-gram model outperformed the CBOW model and the model with vector dimension of 200 performed better than that with smaller number of vector dimensions.

A recent study conducted by Pakhomov and colleagues [22] explored corpus domain effects (i.e., clinical notes, PMC articles, and Wikipedia) on measuring semantic relatedness and similarity between biomedical terms. Clinical notes between 2010 and 2014 obtained from the Fairview Health System were used. Both PMC and Wikipedia datasets included all the data that were available as of September 2015. Modified versions of UMNSRS-Rel and UMNSRS-Sim were used as the reference standards. The CBOW model was used with a fixed window size (i.e., 8) and vector dimension (i.e., 200). Results showed that the model trained on PMC dataset outperformed the one trained on clinical notes (i.e., 0.62 vs. 0.60 for similarity and 0.58 vs. 0.57 for relatedness). It showed the value of publication data on measuring semantic similarity and relatedness between biomedical terms. The effect of corpus size was tested on the dataset of clinical notes and there was no performance increase after the corpus reached a certain size (i.e., 100 M tokens).

Chiu and colleagues [23] did a detailed evaluation of word2vec on biomedical publications by exploring two model architectures and hyper-parameter settings such as negative sample size, sub-sampling, minimum-count, learning rate, vector dimension, and context window size. Their goal was to figure out word2vec’s best-performing settings for each hyper-parameter. Both PubMed and PMC were used as the input corpora. They found that the skip-gram model performed better than the CBOW model and lower-casing of the text corpora achieved better results than without the preprocessing of the original text corpora. Additionally, the best-performing settings for each hyper-parameter were 10 (negative sample size), 1e-4 (sub-sampling), 5 (minimum-count), 0.05 (learning rate), 200 (vector dimension), and 30 (context window size).

As mentioned previously, while these studies’ foci were primarily on the method itself, in this study, we aim to explore how different properties of biomedical publications affect the method’s performance on measuring semantic similarity and relatedness between biomedical terms.

### Data and methods

#### Data

Two sets of data were downloaded from PubMed and PubMed Central (PMC). We downloaded and extracted the abstracts of 18,777,129 articles that were published in the last 30 years (1987–2016) available at PubMed. The smallest number of publications for one year is approximately 360,000 for 1987 whereas more than 1,000,000 for last three years (2014–2016). In addition, 766,326 full-text articles available as of October, 2016 were downloaded from PMC, in which the oldest available full-text article was published in 1896. The number of articles available in the first 30 years (from 1896 to 1927) are 2657 and that of the last 30 years (from 1987 to 2016) are 588, 923. Texts were preprocessed by formatting words into lowercases and tokenizing by the NLTK package [24].

## Methods

### Three aspects of biomedical publication data

1) Recency: Later studies are built from earlier ones and reveal previously unknown knowledge. Thus, our hypothesis is that, with the same amount of research articles, recent articles would convey more meaningful insights on the problem of semantic relatedness and similarity between biomedical terms. To investigate this, we randomly selected 350,000 PubMed abstracts for each of the 30 years to compare the performance of word2vec models trained on 30 distinct datasets. We selected 350,000 as the article size because it is the largest possible size to make sure every year’s dataset includes the same number of research articles.

2) Size: The performance of machine learning models is affected by the size of training datasets. To investigate the size effect, we used stratified sampling to proportionally select PubMed abstracts from the agglomerated 30 years’ dataset that includes 350,000 abstracts each year. We started with 10% of the dataset (which includes 350,000*30*10% = 1.05 million articles) and increased the size with a 10% increment. In other words, we compare the word2vec model trained on 10% of the dataset with the one trained on 20% of the dataset, and so on. We use the dataset that includes 350,000 abstracts for each year rather than the full dataset in order to control the recency effect and make sure that the same number of abstracts are drawn from each year.

3) Section: Abstracts of research articles are known as summaries that include condensed knowledge of whole articles. Researchers usually discuss important findings in abstract sections. We compare the performance of the word2vec model trained on abstracts with the same set of articles trained on the bodies of those articles. Because the PubMed dataset only includes abstracts, we extracted abstracts and bodies separately from the PMC dataset that includes more than 700,000 full-text articles. Both the recency and size effects are under control because the abstracts and bodies are extracted from the same set of articles.

### Performance measures

For evaluations, UMNSRS-Rel and UMNSRS-Sim are used as the reference standards because they are obtained from experts’ judgment [21]. The two reference standards were created by asking eight medical residents to assess the relatedness and similarity of 724 pairs of medical terms. The end results included 587 pairs for the relatedness task and 566 pairs for the similarity task. Medical terms with three semantic types (i.e., disorders, symptoms, and drugs) from UMLS were first selected. Then, a physician complied the terms and included 30 pairs of single-word terms in each of the four relatedness categories (from closely related to completely unrelated). Six semantic types included were disorder-disorder, disorder-symptom, disorder-drug, symptom-symptom, symptom-drug, and drug-drug.

Word2vec models produce cosine similarities of any two given words which ranges from 0 to 1, in which 1 denotes identical and 0 denotes completely different. The reference standards include relatedness and similarity scores of pairs of biomedical terms where a higher value denotes a higher relatedness or similarity. We calculated Spearman’s rank correlations between the reference standards and the results obtained from the word2vec models. Only pairs of biomedical terms whose terms are included in the word2vec models are considered. To do that, we went through every pair of biomedical terms in the reference standards and retrieved cosine similarity of the two terms in the associated word2vec model only if the two words are included in the word2vec model. The word2vec models are trained with the parameter setting recommended by Chiu and colleagues [23] because they reported word2vec’s best-performing settings on biomedical publications after a number of exhaustive experiments.

## Results

### The recency effect

**Fig. 1:**
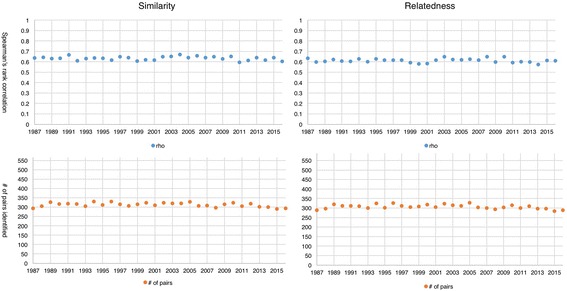
Performance of 30 word2vec models from 1987 to 2016

We trained 30 word2vec models on 30 distinct datasets (each dataset represents a year between 1987 and 2016) with the same data size (each includes 350,000 abstracts). In addition to calculate their correlations with the reference standards, we also recorded the number of semantic relations (relatedness and similarity) identified by each model as shown in Fig. 1. The upper two figures show Spearman’s rank correlations between outputs of the word2vec models and the reference standards and the bottom two figures show the number of relations (i.e., pairs) identified by the word2vec models.

The range of correlations is 0.59 ~ 0.67 for the similarity task and 0.57 ~ 0.66 for the relatedness task. The models successfully identified 291 (51%) to 331 (58%) relations in the similarity task (out of 566 in total in the reference standard) and 284 (48%) to 327 (56%) in the relatedness task (out of 587 in total). The models performed slightly better in the similarity task, but the difference is very small. We performed curve estimations and tested models including Linear, Quadratic, Compound, Growth, Logarithmic, Cubic, S, Exponential, Inverse, Power, and Logistic. All the resulting R-Squares are below 0.1. Overall, we did not find any evidence that models trained on recent articles perform better than their counterparts. Thus, we can safely conclude that there is no recency effect. One noticeable fact is that these models were only able to identify roughly half of all pairs in the reference standards (i.e., 331/566 for similarity and 327/587 for relatedness). This was probably due to the relatively small size of the dataset, and we investigate how data size affects model performance in the next section.

### The size effect

**Fig. 2:**
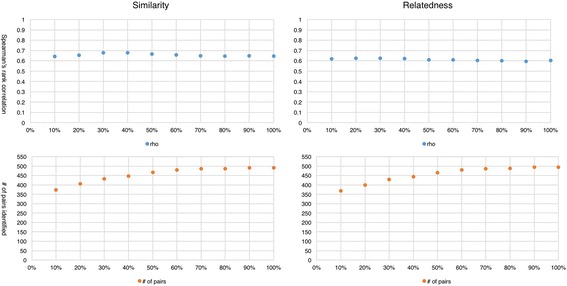
Performance of 10 word2vec models from 10% to 100%

Ten models were trained separately by systematically increasing the size of the PubMed dataset from 10% to 100%. We used stratified sampling to ensure that data extracted from every year make an even contribution to the final dataset. Spearman’s rank correlations and identified numbers of relations are shown in Fig. 2.

As shown in Fig. 2, for the similarity task, the range of correlations is from 0.64 to 0.68 and the range of identified numbers of pairs is from 374 (66%) to 491 (87%). For the relatedness task, the range is from 0.59 to 0.63 and from 368 (63%) to 494 (84%), respectively. In terms of correlations, the models performed better in the similarity task, which corresponds to the result obtained in the previous experiment. We found that as the size of the datasets increases, the models identified more pairs of biomedical terms. Table 1 shows the number of distinct vocabularies in each of the 10 datasets and the number of identified pairs of biomedical terms in each task. We see that more relations were identified as the size of distinct vocabularies increase. Another important finding is that, in both tasks, the performance (i.e., correlations with the reference standards) reached the highest at the point of 30% and began to decrease notably at the point of 40%. This finding aligns with the one made by Pakhomov and colleagues [22], where they also reported that there is no performance gain beyond a certain point. In our experiment, as shown in Fig. 2, the point is 4 million distinct vocabularies.

**Table 1:** Number of distinct vocabularies in each dataset

Percentage	# of vocabularies	# of pairs identified (sim)	# of pairs identified (rel)
10%	1,451,218	374 (66%)	368 (63%)
20%	2,339,000	406 (72%)	399 (68%)
30%	3,313,239	432 (76%)	429 (73%)
40%	3,961,051	447 (79%)	444 (76%)
50%	4,572,957	467 (83%)	465 (79%)
60%	5,319,879	479 (85%)	480 (82%)
70%	5,856,126	486 (86%)	487 (83%)
80%	6,369,803	486 (86%)	488 (83%)
90%	7,016,215	491 (87%)	494 (84%)
100%	7,797,722	491 (87%)	494 (84%)

### The section effect

We extracted abstracts and bodies of articles separately from the PMC dataset that includes more than 700,000 full-text articles. Based on the extracted abstracts and bodies of texts, we trained two word2vec models. The test result is shown in Table 2.

**Table 2:** Performance of word2vec models trained on abstracts and bodies

Section	Similarity		Relatedness	
	Correlation	# of pairs	Correlation	# of pairs
Abstract	0.65	344 (61%)	0.66	339 (58%)
Body	0.62	498 (88%)	0.59	503 (86%)

As shown in Table 2, in both relatedness and similarity tasks, the model trained on abstracts performed better than the one trained on bodies, i.e., 0.65 vs. 0.62 in the similarity task and 0.66 vs. 0.59 in the relatedness tasks. On the other hand, in both tasks, the model trained on bodies identified more pairs of biomedical terms than its counterpart. To further examine any statistical differences, we trained 20 word2vec models by dividing each section into 10 equal groups and performed t-tests. In the similarity task, models trained on abstracts showed greater correlations with the reference standards than the ones trained on bodies (0.55 vs. 0.34), but identified fewer pairs of biomedical terms (79 vs. 196). Both are significant at an alpha level of 0.05. Similar results were obtained in the relatedness task. Models trained on abstracts performed better in correlation (0.44 vs 0.25) with the reference standards, but worse in the number of identified pairs of biomedical terms (78 vs 197) at a significance level of 0.05. This is expected because bodies include more vocabularies than abstracts. However, this also makes bodies of articles include more irrelevant terms than the abstracts and leads to worse performance. There is a trade-off between accuracy and the identifiable size of relations. Table 3 shows two sets of top 10 pairs with the highest similarity in the model trained on bodies, which were not identified by the model trained on abstracts. Top 10 pairs in both tasks are listed. There are a few duplicate pairs as the reference standards do.

**Table 3:** Top 10 pairs identified only by the model trained on bodies

Rank	Similarity			Relatedness		
	Term 1	Term 2	Cosine sim.	Term 1	Term 2	Cosine sim.
1	xanax	ativan	0.76	xanax	ativan	0.76
2	spiriva	serevent	0.75	zoloft	prozac	0.73
3	zoloft	prozac	0.73	pepcid	zantac	0.71
4	tylenol	motrin	0.72	atacand	avapro	0.71
5	pepcid	zantac	0.71	actonel	fosamax	0.69
6	actonel	fosamax	0.69	medrol	prednisolone	0.68
7	medrol	prednisolone	0.68	cardura	hytrin	0.67
8	cardura	hytrin	0.67	albuterol	serevent	0.63
9	meningism	hyperesthesia	0.66	photophobia	meningism	0.62
10	cozaar	diovan	0.65	mycosis	blastomycoses	0.61

As shown in Table 3, among the 26 unique terms, five (i.e., *blastomycoses*, *hyperesthesia*, *meningism*, *mycosis*, and *photophobia*) are disease names and two (*albuterol* and *prednisolone*) are general names of drugs, and 19 terms are brand names of drugs. We can see that authors tend not to mention brand names of drugs in abstracts and this is one of the major reasons that made the model trained on abstracts only able to identify a limited number of pairs. Overall, abstracts are good for producing more accurate results whereas bodies of articles should be used if comprehensiveness matters.

Overall, we evaluated the effects of recency, size, and section from two perspectives: correlation with the reference standards and the number of identified pairs of biomedical terms. Each measure has its own advantages and disadvantages. The correlation-based evaluation mainly focuses on accuracy while the latter puts more emphasis on comprehensiveness. A specific word2vec model may boast its high accuracy with only a limited number of identified pairs of biomedical terms and vice versa. Therefore, these two measures should be used together to get more insights. We used the two reference standards in the evaluation: relatedness and similarity; however, there is no such a differentiation in word2vec. Even though word2vec produces similarity scores for any pairs of words, these scores could be represented as either degree of relatedness or similarity.

## Discussion and conclusions

In this study, we investigated the performance of the word2vec models trained on PubMed and PMC in identifying semantic relatedness and similarity of biomedical terms. This study is the first that tackled the problem from the data perspective—we examined the effects of recency, size, and section of publication data on the performance of the word2vec models. Results showed that models trained on recent datasets did not boost the performance. Additionally, we found that models trained on larger datasets identified more pairs of biomedical terms of semantic relatedness and similarity than models trained on smaller datasets. The largest dataset identified 100 more pairs of biomedical terms (out of more than 500 pairs) than the smallest dataset. In addition, the highest correlation with the reference standards was obtained at the point of 3.3 million distinct vocabularies and there was a notable decrease after the vocabulary size reached 4 million. Another important finding is that the model trained on abstracts produced results that have higher correlations with the reference standards than the one trained on article bodies. However, the latter identified 150 more pairs of biomedical terms than the former.

We conclude that increasing the size of dataset does not always enhance the performance. There is a certain point at which the performance reaches the highest level and more noise starts to comprise the performance as more data are added. This finding is in line with the one reported in the study by Pakhomov and colleagues [22] in which they found that there is also such a point in the dataset of clinical notes that the performance started to decline after the size of the publications reached to that point. Second, increasing the size of datasets can result in the identification of more relations of biomedical terms even though it does not guarantee better precision. Third, as summaries of research articles, compared with article bodies, abstracts excel in accuracy but lose in coverage of identifiable relations. Last, the performance of tested word2vec models did not exceed a certain level, i.e., a 0.7 correlation, with the reference standards. Thus, word2vec models trained on publication data may have limitations in some biomedical applications where high levels of accuracy are required. However, in applications such as query term suggestion and query expansion where a moderate accuracy is sufficient, the strengths of word2vec models are evident.

There are a few limitations in the study. First, even though the reference standards used in the study have been used by a few previous studies, they do not represent the only gold standard. Therefore, using other reference standards may produce different results. Second, because there are possible different parameter settings and implementations of the word2vec technique, slightly different results may be obtained if different parameter settings or implementations are used. Last, we did not conduct cross-domain comparisons because the word2vec technique has not been applied to other disciplinary corpora for the purpose of evaluating semantic relatedness and similarity of biomedical terms.

Given its decent performance reported by multiple studies, word2vec will be continuously used by biomedical applications to take advantage of the rich information embedded in ever-increasing biomedical scientific publications. Findings of this study help biomedical researchers apply word2vec in a more informative way and help them improve the method’s performance on extracting relatedness and similarity information from biomedical publication data.
